# Public Perceptions of Marital Rape: Does Level of Force Used Have an Impact?

**DOI:** 10.1002/bsl.70036

**Published:** 2026-01-10

**Authors:** Leanne Hanney, Amy Shelford, Andy Guppy

**Affiliations:** ^1^ Arden University London UK; ^2^ University of Bedfordshire Luton UK

**Keywords:** attitudes towards women, domestic violence, marital rape, perceptions of rape, rape myth acceptance

## Abstract

Research indicates that marital rape is viewed by the public as less harmful to a victim than stranger/acquaintance rape. The aim of the study is to extend the research conducted by Robinson in 2017, investigating how levels of force influence perceptions of marital rape. The study also examines how rape perception is influenced by rape myth acceptance and attitudes towards women. The current study improves on previous work by controlling for individual differences across groups using a repeated‐measures design. The results indicate that as the level of force increases the perception of marital rape increases. Positive attitudes towards women and low rape myth acceptance are also found to have a positive impact on perceptions of marital rape. Based on these findings, it is possible to recommend that further awareness of legislation regarding coercion and marital rape is required within the public domain.

## 1 Introduction

Rape can be defined as unwanted sexual penetration committed via force, threat of physical harm or when the victim cannot provide consent due to intoxication or mental impairment (West 2002). According to the World Health Organization (2021), 1 in 3 women worldwide were victims of physical or sexual violence between 2000 and 2018. The Crime Survey for England and Wales determined that 1.1 million adults over the age of 16 had been victims of sexual assault in the UK in the year ending March 2022 with a total of 193, 566 sexual offences recorded (Office for National Statistics 2023). When reviewing the victim's relationship to the offender, the Office for National Statistics (2023) identified that 5.7% of adults have been victims of sexual assault from a partner or family member since the age of 16 years and that 4.9% of adults were victimised by their partners since the age of 16.

Despite statistics highlighting the prevalence of rape, members of the public hold stereotypes around the subject which Burt (1980) states have resulted in hostility towards victims of rape. These stereotypes are known as rape myths and include the act of denying or reducing the injury of a rape victim and blaming the victim for their own victimization. Burt (1980) investigated attitudes which were likely to predict rape myth acceptance (RMA) and determined that there are high levels of RMA for individuals who have high levels of sex‐role stereotypes, adverse sexual beliefs, and acceptance of interpersonal violence. However, lower levels of RMA were found in younger and better educated individuals who also showed low levels of stereotypes, adverse sexual beliefs, and proviolent attitudes. Furthermore, Suarez and Gadalla (2010) performed a meta‐analysis of 37 studies published between 1997 and 2007 which determined that males were more likely to endorse RMA. The analysis also identified a strong positive link between sexual aggression/ hostile/ aggressive attitudes towards women and RMA. However, Suarez and Gadalla (2010) have argued that the analysis is limited because a variety of measures were used to assess attitudes and behaviours, including tools created specifically for the single study. Additionally, Russell and Hand (2017) conducted a rapid evidence assessment which identified that rape myth acceptance is more common within males, and that victim blaming was attributed more to stranger rape scenarios rather than acquaintance rape. However, included sources may be limited in rapid evidence reviews, and prone to bias (Leeuw and Schmeets 2016). With only four empirical studies used in the Russell and Hand (2017) rapid evidence assessment, this limitation must be taken into consideration before drawing conclusions about the influence of gender or situational factors on RMA. An additional limitation to consider is that many RMA scales have not been psychometrically tested for gender invariance and that some research has identified that not all items are invariant across genders (Fakunmoju et al. 2019). This implies that attempting to compare between male and female respondents can be difficult and can impact the findings.

Research implies that sexist beliefs and hostile attitudes towards women may have significant effects on RMA (Suarez and Gadalla 2010; Angelone et al. 2015; Hill and Marshall 2018; Rollero and Tartaglia 2019). In addition to this, research has also investigated sexist beliefs as a mediator between gender and RMA found supporting evidence suggesting that males were more likely to have higher levels of RMA than females (Angelone et al. 2021).

### 1.1 Marital Rape

One specific rape myth implies that husbands cannot rape their wives (Edwards et al. 2011; C. Lilley et al. 2023). Marital rape has been defined as non‐consensual sexual activity performed by one legal spouse to another (Simonson and Subich 1999; Utami and Haeranah 2022). In the UK in 1992, marital immunity regarding rape was rebuked due to the case R v R (1991) where the House of Lords came to the decision that in modern times the husband's immunity was no longer part of English law (The Law Commision 1992). Despite this legal change, victims of marital rape are still treated differently to other victims of rape and are required to report incidents of rape in a shorter time‐period, as well as being expected to have experienced force or the threat of force (Barkan and Bryjak 2011).

Research has found that victims of marital rape suffer negative physical and psychological impacts (Tarzia 2021; C. Lilley et al. 2023). This includes low self‐esteem, negative feelings towards men, suicide attempts, substance use disorders, miscarriages, rectal bleeding, vaginal bleeding, along with other symptoms (Filip and Popp 2021; Agarwal et al. 2022; C. Lilley et al. 2023). In a clinical respect, victims of marital rape are more likely to develop cervical cancer, cervical dysplasia, an increased risk of suicide, and a risk of becoming victims of homicide (Martin et al. 2007). Yet despite experiencing these physical and psychological symptoms, some victims of marital rape do not seek help (Martin et al. 2007). Reasons for this may be that some victims fear that they will not be believed, fear not being taken seriously by police, being blamed, being rejected by society, they don't perceive the incident as rape, or fear potential repercussions from their husbands, as well as other reasons (Filip and Popp 2021; Sharma and Mishra 2021; C. Lilley et al. 2023). These negative outcomes highlight the extensive and complex difficulties that victims of marital rape face, showing the need for research that reviews the public understanding of the severity of marital rape, and the need for greater awareness.

### 1.2 Public Perceptions of Marital Rape

Simonson and Subich (1999), investigated the public perceptions of rape in relation to the association between the victim and the perpetrator in the USA. The results identified that the marital rape scenario was less frequently classified as rape by participants in comparison to the stranger, acquaintance, and date rape scenarios. In addition to this, marital rape was viewed as less violent and less psychologically damaging. Frese et al. (2004), reported supporting findings using a Spanish participant pool. Ferro et al. (2008) also found similar results when comparing rape myth acceptance for marital rape to acquaintance rape in the USA. This suggests that perceived levels of victim‐offender relationship within rape scenarios may impact the perception of rape. Kuriakose (2019) replicated the study by Ferro et al. (2008) and compared attitudes of young adults in the U.K. and India. The results showed that participants who read the marital rape scenario were more likely to support the false myth regarding marital rape than those who read the general rape scenario. However, the participants did not assign blame to the victim regardless of the scenario. The study also identified a cultural difference in perceptions of rape where U.K. participants were more likely to see the act as deviant than Indian participants. Kuriakose (2019) argues that this is due to marital rape being illegal in the U.K. but not classified as a criminal act in India.

It is argued that the acceptance of the myths surrounding marital rape, and poor attitudes towards women, have a negative impact on societies acknowledgement and response to the issue (Mahoney and Williams 1998). It leads to poor mental and physical health outcomes for the individual, subsequent unnecessary strain on healthcare services, contributes to the victim's perceived stigma and fears of judgement, and perpetuates rape myth acceptance culture through the resultant low rates of reporting marital rape (Martin et al. 2007; Woolley 2007; Agarwal et al. 2022). Further research is needed to determine the influences on the relationship between rape myth acceptance, attitudes towards women, and perception of rape, such as perceived level of violence, so that future work can begin to challenge and ameliorate public misconceptions of marital rape and alleviate the burden it has on individuals and society.

### 1.3 The Impact of Levels of Force in Rape Scenarios

Another factor to consider when reviewing perceptions of marital rape is the amount of force used by the perpetrator. Home Office (2022) defines coercion as a form of domestic abuse which involves the use of violence, threats, humiliation or psychological intimidation to control a victim and make the victim do things which they do not want to do. This usually coincides with other abusive behaviours such as physical, sexual and financial abuse. Research has indicated that sexual coercion occurs across a continuum ranging from engaging in unwanted sex in return for behaviours (i.e., being brought dinner) to use of physical force (Basile 2002; Oswald and Russell 2006; DeGue et al. 2010; Viejo and Sanz 2020).

With the aim of understanding public perceptions of coercive control, Lagdon et al. (2023) asked participants to review obvious coercive behaviour (taking control of finances to prevent the partner from travelling without permission) and less obvious coercive behaviour (asking the partner to cancel plans with friends and family if the controlling partner cannot be there) within heterosexual romantic couples. The results indicated that many participants did not recognize less obvious forms of coercive control which suggests the need for greater awareness of behaviour which can indicate a risk of intimate partner violence (Lagdon et al. 2023). Robinson (2017) conducted a study which investigated how the level of force used by an offender could impact the perceptions of marital rape. Participants were randomly assigned to one of three vignettes, each showing different levels of force in a marital rape scenario. The results indicated that participants who read the high level of force scenario (violence) were more likely to perceive the incident as rape than those in the in the low force (ignoring the lack of consent) or medium force (pinning the victim) conditions. There was also a significant difference between genders, in support of previous research. The results also suggest that the increase of rape myth rejection was associated with higher perceptions of marital rape and that females were more likely to reject rape myths than males. There was a non‐significant interaction between attitudes towards women's roles in society or rape myth rejection and the level of force conditions (Robinson 2017). However, the study by Robinson (2017) can be critiqued because of the use of an independent‐group design. An independent‐groups design isolates each group's results making it difficult to determine if attitudes changed when the level of force changed or if this was a confound of the original study (Evans and Rooney 2008). A repeated‐measures design allows the researchers to solely investigate the impact of the change in level of violence on the perception of rape in a marriage, controlling for the influence of individual differences in experience and attitudes on the perception of marital rape (Shoda 2004). In addition to this, Robinson (2017) can also be critised for the use of participants from a college designed to teach courses specialized in criminal justice. These courses may have already provided the participants with knowledge about rape myths before data collection and unintentionally causing a priming effect.

### 1.4 The Current Study

Previous research indicates that marital rape is perceived to be less harmful to the victim than stranger/acquaintance rape (Simonson and Subich 1999; Ferro et al. 2008; Kuriakose 2019), despite the research findings which demonstrate the physical and psychological impact on victims (Martin et al. 2007; Filip and Popp 2021; Tarzia 2021; C. Lilley et al. 2023). The current study aims to replicate and extend the research conducted by Robinson (2017) to investigate how levels of force influence perceptions within marital rape, and how perception of force is influenced by rape myth acceptance and attitudes towards women. This study extends previous research by utilising a repeated measures design to reduce error variance and by also incorporating individual differences in terms of gender, rape myth acceptance and attitudes towards women. This study will also attempt to control for the potential priming effects within Robinson (2017) by recruiting participants from social media and by counterbalancing the vignettes. For the current study, marital rape will focus on non‐consensual sexual activity between one legal spouse and another.

The research question proposed asks: does the perception of marital rape differ with the perceived level of force and relevant attitudes towards women and rape myth acceptance?

1. It is hypothesised that the perceived likelihood of rape will increase as the level of force described in the scenarios increases.
2. Lower rape myth acceptance scores will be associated with greater perception of rape in scenarios.
3. More positive attitudes towards women will be associated with greater perception of rape across scenarios.

## 2 Method

### 2.1 Design

An online survey incorporating a mixed (within‐subjects and between groups) ANOVA design was used. The within‐subjects factor was degree of force with three levels used across the rape scenarios: subtle, moderate and extreme force (see Supporting Information S1: Appendix 2). The dependent variables were the participant's perceptions of marital rape taken from their scores on the post‐vignette survey for each scenario (see Supporting Information S1: Appendix 1). The use of a within‐participants design is applied due to its ability to focus on the impact of the change in independent variable, and its ability to control factors such as individual differences by requiring the participants to complete all conditions of a study (Hellier 1998; Shoda 2004). However, an existing limitation to the within‐participants design is the risk of order effects which allows participants to learn and improve on their previous experiences (Brown and Sleath 2016). To control for this, counterbalancing will be applied to the vignettes paired with their appropriate post‐vignette surveys. The between groups factors involved in the present paper were gender, attitudes towards women and rape myth acceptance.

### 2.2 Participants

An a priori power analysis was conducted using G*Power version 3.1.9.7 (Faul et al. 2007). To achieve an 95% power, for identifying a medium effect size (*f* ^2^ = 0.25; Cohen 1988) with *α* = 0.05 for 3 measures, across 8 groups the minimum participant *N* = 48 for an a priori power analysis for repeated‐measures ANOVA.

One hundred and twenty‐two respondents took part in this study; however, after removing incomplete questionnaires from the data, *N* = 99 surveys were completed. A total of 63.6% were female, 65.7% = Caucasian, 76.8% = single and 53.5% of the participants were within the age range of 18–25 years old.

### 2.3 Materials

Three marital rape vignettes created by Robinson (2017) in collaboration with experts working with sexual assault victims and offenders were used. Each depicted a scene where a married heterosexual couple engage in non‐consensual sex. In the vignettes the female refuses the male's sexual advances and the male proceeds with intercourse. However, the level of force used by the male differed in each vignette (see Supporting Information S1: Appendix 2). For this study, the names of the individuals in the vignette and the locations, have been changed to reflect 3 different heteronormative couples living in different cities in the UK with the aim of improving generalizability and imitating the diversity of the real world (Devine and Heath 1999; Agostini et al. 2024).

Post‐vignette surveys from the replicated study (Robinson 2017) included 14 questions about the scenario and the responses were recorded using a four to six‐point Likert‐scale. The survey was created by Robinson (2017), based on an existing measure originally developed by Simonson and Subich (1999). This was presented to the participants after each vignette and modified to include the names used the vignette they had just reviewed (Supporting Information S1: Appendix 1).

The Attitudes Towards Women Scale—Short Survey (Spence et al. 1973) contains 25 questions measured using a Likert scale where 1 = strongly agree and 4 = strongly disagree. Collated scores reflect attitudes towards women, where lower scores indicate a more traditional view of women's role in society. To assess the internal consistency of the ATWS‐SS (Spence et al. 1973) a reliability analysis was run using the participants from the current study. The scale received a Cronbach's Alpha value of *α* = 0.90.

The Updated Illinois Rape Myth Acceptance scale (McMahon and Farmer 2011) contains 22 questions measured using a Likert scale, where 1 = strongly agree and 5 = strongly disagree. Collated scores reflect rape myth acceptance with higher scores reflecting higher rejection of rape myth. A reliability analysis was performed to assess the internal consistency of the UIRMAS (McMahon and Farmer 2011) and a Cronbach's Alpha value of *α* = 0.96 was received using the current study's participant responses.

The materials were combined and distributed in an online survey format using the Qualtrics XM platform.

### 2.4 Procedure

The current study followed the procedure outlined in Robinson (2017) with some minor changes.

Participants of all backgrounds over 18 years old were recruited online through opportunity and snowball sampling, by sharing online links on Facebook, Twitter, Instagram, and LinkedIn between 21st June 2023 and 12th July 2023, as well as asking for it to be shared on. Recruitment messages, information and consent forms, and the Qualtrics skip logic were used to discourage and prevent participation from victims of sexual violence as well as anyone under the age of 18. This was with the intention of reducing the risk of revictimisation and to prevent harm to individuals under the age of 18 based on the ethical principles outlined by the British Psychological Society (Oates et al. 2021).

At the beginning of the study, participants were told that the study is investigating perceptions of sexual violence. The information sheet outlined their participation, as well as their rights as a participant, including anonymity, confidentiality, protection from harm, and their right to withdraw. Participants were then presented with a consent form. Victims of sexual violence, and prospective participants under the age of 18 were excluded from the study through dissuasion on the information sheet and skip logic in the consent form questions.

The participants then completed the demographic questionnaire, The Attitudes Towards Women Scale—Short Survey ([ATWS‐SS] Spence et al. 1973), and the Updated Illinois Rape Myth Acceptance Scale ([UIRMAS] McMahon and Farmer 2011). These were counterbalanced with the presentation of all three marital rape vignettes (adapted from Robinson 2017), along with all three post‐vignette surveys adapted to reflect the names in the preceding vignette (Robinson 2017). Counterbalancing was utilised to control for sequencing effects by presenting the scenarios and measures of attitudes in a random order (Edmond and Kennedy 2016). Each of these scenarios are fictitious and differ in the described level of force used.

Upon completion of the study, the participants were provided with a debrief form which explained the full nature of the study and thanked the participants for their time. This also signposted participants to relevant support services if they felt that they had been affected by the research topic and provided the contact details for the researchers should they wish to withdraw their data, request a summary of the results, or make a complaint. No compensation or incentive was offered to participants to complete this study.

The study was designed and conducted in line with the British Psychological Society Code of Ethics (Oates et al. 2021). Approval for this study was granted by the University of Bedfordshire School of Psychology ethics committee.

## 3 Results

### 3.1 Descriptive Statistics

Table 1 displays the descriptive statistics of the participants who successfully completed the survey. The majority of participants were single, Caucasian, female, and within the age range of 18–25 years old.

**TABLE 1:** Participant descriptive statistics.

Gender	Frequency (*N*)	Percentage (%)
Female	63	63.6
Male	35	35.4
Transgender	1	1.0
Total	99	100
Age range (years)		
18–25	53	53.5
26–35	28	28.3
36–45	6	6.1
46+	12	12.1
Ethnicity		
Caucasian	65	65.7
Hispanic/Latino	1	1.0
Black/African American	3	3.0
Asian	24	24.2
Other	6	6.1
Marital status		
Single	76	76.8
Married	16	16.2
Living in common law	3	3.0
Separated	1	1.0
Divorced	2	2.0
Widowed	1	1.0

To identify whether gender, ATWS‐SS scores and UIRMAS scores had an impact on the participants' perception of rape in each condition a mixed ANOVA was run. The within‐subjects factor was levels of forces with each respondent providing a rating of each of the three scenarios. Table 2 shows the mean rape perception scores for the gender, ATWS‐SS and the UIRMAS groups across each scenario when responding to question 11 of the post‐vignette survey (Would you characterize this intercourse as rape?). In both the subtle and moderate force scenarios, the highest mean scores were observed in those participants who had high ATWS‐SS scores and low UIMAS scores (mean subtle force ratings males = 5.71, and females = 5.41 and mean moderate force ratings male = 5.71, and female = 5.72). However, for the severe force scenario, the highest mean scores were observed in participants who had high ATWS‐SS scores and high UIRMAS scores (mean severe force ratings males = 6.00 and females = 5.92).

**TABLE 2:** The mean rape perception scores for low and high ATWS‐SS and UIRMAS groups for each gender across each level of force.

Level of force	ATWS‐SS^a^	UIRMAS^b^	Gender	Mean	SD	*N*
Subtle force	Low	High	Male	4.52	1.36	21
			Female	3.85	1.67	13
		Low	Male	5.33	1.15	3
			Female	4.90	0.73	10
	High	High	Male	4.25	1.50	4
			Female	5.00	1.20	12
		Low	Male	5.71	0.48	7
			Female	5.39	0.95	28
Moderate force	Low	High	Male	4.81	1.50	21
			Female	4.31	1.43	13
		Low	Male	5.33	1.15	3
			Female	5.40	0.84	10
	High	High	Male	5.50	0.57	4
			Female	5.58	1.44	12
		Low	Male	5.71	0.75	7
			Female	5.71	0.60	28
Severe force	Low	High	Male	5.00	1.14	21
			Female	5.08	1.11	13
		Low	Male	5.00	1.73	3
			Female	5.70	0.48	10
	High	High	Male	6.00	0.00	4
			Female	5.92	0.28	12
		Low	Male	5.71	0.75	7
			Female	5.89	0.31	28

^a^ Attitudes Towards Women Scale—Short Survey.

^b^ Updated Illinois Rape Myth Acceptance scale.

### 3.2 Main and Interactions Effects (Within‐Subjects)

Table 3 summarises the Within‐Subjects main and interaction effects from the ANOVA. There was a significant effect of levels of force found (*F* = 14.97, df 2, 180, *p* < 0.001, partial *η* ^2^ = 0.14) and from the means in Table 2, there was a steady increase in the rape perception scores as scenario force increased. There was also a significant interaction between Rape Myth groups and Force conditions (*F* = 5.95, df 2, 180, *p* = 0.003, partial *η* ^2^ = 0.06). Examination of the group means suggests that while there was only a small increase in rape perceptions across the force scenarios for the low Rape Myth Acceptance group, there was a much larger impact on rape perceptions for the high Rape Myth Acceptance group as force increased. There were no other significant Within‐Subjects effects.

**TABLE 3:** Within‐Subjects effects for perception of rape in each force condition by ATWS‐SS, UIRMAS and gender groups.

Effects	*F*	df	*p* (2‐tailed)
Force	14.97***	1.92, 173.38	< 0.001
Force × ATWS‐SS^a^	0.62	1.92, 173.38	0.53
Force × UIRMAS^b^	5.94**	1.92, 173.38	< 0.01
Force × gender	1.37	1.92, 173.38	0.25
Force × ATWS‐SS^a^ × UIRMAS^b^	0.87	1.92, 173.38	0.41
Force × ATWS‐SS^a^ × gender	2.53	1.92, 173.38	0.08
Force × UIRMAS^b^ × gender	1.64	1.92, 173.38	0.19
Force × ATW‐SS^a^ × UIRMAS^b^ × gender	0.49	1.92, 173.38	0.60

^a^ Attitudes Towards Women Scale—Short Survey.

^b^ Updated Illinois Rape Myth Acceptance scale.

*p < 0.05.

^**^ *p* < 0.01.

^***^ *p* < 0.001.

### 3.3 Between‐Subjects Effects

Table 4 outlines the Between Subjects effects from the ANOVA. It can be seen that there were significant main effects for the UIRMAS (*F* = 4.64, df 1, 90, *p* = 0.034, partial *η* ^2^ = 0.05) and ATWS‐SS (*F* = 6.62, df 1, 90, *p* = 0.012, partial *η* ^2^ = 0.07) groups. The groups means indicated that more positive attitudes to women and low Rape Myth Acceptance were associated with higher rape perceptions. There was no significant main effect of gender (*F* = 0.00, df 1, 90, *p* = 0.954, partial *η* ^2^ = 0.00) and none of the Between Subjects interaction effects were significant.

**TABLE 4:** Between‐Subjects ANOVA effects for the mean rape perception score by ATWS‐SS, UIRMAS and gender groups.

Effects	*F*	df	*p* (2‐tailed)
ATWS‐SS^a^	6.62**	1, 90	0.01
UIRMAS^b^	4.63*	1, 90	0.03
Gender	0.03	1, 90	0.95
ATWS‐SS^a^ × UIRMAS^b^	0.63	1, 90	0.43
ATWS‐SS^a^ × Gender	0.24	1, 90	0.62
UIRMAS × Gender	0.03	1, 90	0.84
ATWS‐SS^a^ × UIRMAS^b^ × Gender	0.69	1, 90	0.40

^a^ Attitudes Towards Women Scale—Short Survey.

^b^ Updated Illinois Rape Myth Acceptance scale.

^*^ *p* < 0.05.

^**^ *p* < 0.01.

***p < 0.001.

## 4 Discussion

### 4.1 Levels of Force and Perceptions of Marital Rape Interactions

The present study aimed to examine whether the perception of marital rape would differ as the perceived levels of force altered. The current study identified that the perceptions of marital rape were different among levels of force in each scenario. These results indicated that as the level of force used in each scenario increased, the perception of marital rape increased and support the previous findings of Robinson (2017). These findings also support the outcomes of similar studies which focussed on how the use of force and the level of that force can impact perceptions of rape (Krulewitz and Payne 1978; Vandiver and Dupalo 2013). It is possible to argue that the current findings imply that individuals are better at perceiving a situation as marital rape when a severe level of force is used. Previous research has highlighted that perceived severity is influenced by both details in vignettes and the participants endorsements of rape myths (Leon and Aizpurua 2024; Serrano‐Montilla et al. 2025). Therefore, it can be suggested that there is a need for improved public awareness regarding the legislation relating to rape, coercion, and marital rape.

### 4.2 RMA, Attitudes Towards Women and Levels of Force Interactions

The present research aimed to identify whether a lower RMA score would predict a higher perception of rape score. The results from the current study suggest that there was a significant main effect for the UIRMAS factor. This indicates that lower scores on the UIRMAS are associated with higher perception of marital rape and supports the hypothesis; however, this disputes the findings of Robinson (2017). There was also a significant Force × RMA interaction that suggested that while low RMA participants reported higher ratings of rape perception across the scenarios, there was a sharper increase of rape perception for high RMA participants as force increased. Burgin (2019) has suggested that individuals rely on the use of physical force within an event to determine if it is classified as rape and this can negatively impact people's understanding of the severity of the situation. This is due to people endorsing the myth that for something to be rape, it must involve physical violence (Jenkins 2021). Therefore, it is possible to argue that the increase in rape perception for participants with high RMA which occurred as force increased in the current study could be due to their understanding of traditional rape scenarios. This would indicate a need for interventions to increase public awareness of how the lack of consent, and the use of coercion are also legally classified as acts of rape in the UK.

The current research also aimed to examine if more positive attitudes towards women would predict a higher level of rape perception. The results found a significant main effect for ATWS‐SS which indicated that higher ATWS‐SS scores are associated with higher perceptions of marital rape. However, these results dispute the findings of Robinson (2017). It is possible to argue that the current findings regarding the UIRMAS and ATWS‐SS scores disagree with the results of Robinson (2017) due to some of the differences in research design. In the original paper, participants were selected from college students in New York and the vignettes described couples who lived in New York, which may have impacted the results (Robinson 2017). In addition to this, the participants included in the research by Robinson (2017) were students studying at a college which focussed on teaching courses which specialized in criminal justice, these courses may have made the participants aware of the issues surrounding rape myths prior to data collection. Whereas, in the current study the participants were conveniently sampled through social media, allowing for a wider range of nationalities to participate. Additionally, the vignettes were set in a variety of English cities with different ethnic couples in an attempt to improve generalizability of the findings. It can also be argued that the current study has found different results because Robinson (2017) supplied participants with the UIRMAS and ATWS‐SS prior to the vignettes, which may have caused priming effects. However, the current study counterbalanced the attitude surveys and vignettes to reduce potential socially desirable responses and priming effects.

It should also be noted that since the publication of the Robinson (2017) study, there has been a shift in some public attitudes after the #metoo movement went viral in October 2017. This movement has highlighted the prevalence of sexual violence worldwide and a greater need for better systems to support survivors (me too. Movement 2025). Research has identified that this shift in social context has influenced an increase in public awareness of unwanted sexual experiences as incidents of sexual assault (Szekeres et al. 2020; Jaffe et al. 2021). Therefore, it is possible to argue that this global cultural shift can explain the differences between attitudinal findings from Robinson (2017) and the current study.

### 4.3 Participant Gender as a Factor of Interest

The present study also included an analysis of the data after classifying gender as a factor of interest. As there was only one participant who described themselves as transgender, their data was removed from the analysis. These results showed that the perceptions of marital rape remained significant among levels of force in each scenario. In addition to this, a significant effect for the UIRMAS total scores was found, as well as a significant effect for the ATWS‐SS total scores. However, there were no significant effects for gender which suggests that gender has no association to perceptions of marital rape. This differs from previous research that indicates the existence of a relationship (Suarez and Gadalla 2010; Russell and Hand 2017; Robinson 2017; Angelone et al. 2021). A further exploration of the present results revealed a significant simple effect for gender only on the responses to the most severe scenario and not on the others. Furthermore, this simple effect became non‐significant with the addition of ATWS‐SS and UIRMAS scores. This indicates that the impact of gender in the present study was only linked to the severe scenario and even this disappeared once relevant attitudes and beliefs were considered. It is possible to argue that no significant interaction was found for gender × UIRMAS × ATWS‐SS in the current study because previous research has found general sexist attitudes to act as a mediator for RMA regardless of gender (Angelone et al. 2018; Angelone et al. 2021). Angelone et al. (2021) has argued that both men and women can hold sexist attitudes, and that future research should review the different subcategories of sexism, such as benevolent sexism to understand this further. It can also be argued that the findings from the current study may differ from previous research due to the cultural shifts that have occurred in western culture over the past 50 years Eagly and Mladinic (1989) argued that the ATWS‐SS measured attitudes related to women's equal rights rather than attitudes towards women. In response to this critique, Spence and Hahn (1997) used the ATWS‐SS to review cohort changes in college students between 1972, 1976, 1980 and 1992. The results identified that both male and female attitudes towards women were becoming more pro‐feminist. It should also be noted another large cultural shift took place globally in 2017 cause by the #metoo movement. Research has shown that there has been a decrease in dismissive attitudes towards rape and sexual violence regardless of gender since this cultural shift; however, this is also mediated by other factors such as political stance (Szekeres et al. 2020).

### 4.4 Limitations

It can be argued that the current study is limited due to the participant sample being predominantly young female Caucasians. These population factors make it difficult to demonstrate generalizability to the wider population because the sample from the current study may share similar attitudes towards a particular topic (Walker et al. 2010). To overcome this limitation, future research could use a purposive sampling technique with the aim of creating more variation within the participant pool (Stommel and Wills 2004).

The current study can also be critiqued for this use of social media to recruit participants. C. Wilson (2024) argues that social media has a built‐in bias where potential participants usually share demographics with the researcher who has posted the advert. This can limit the generalizability of the results. To overcome this limitation, C. Wilson (2024) suggests that researchers should include a screening phase to a study so that participants can be thoroughly checked by researchers prior to data collection or by utilizing a data collection service where people are paid to take part in large research studies.

Another methodological limitation to consider for the current study is the use of vignettes. Vignettes can be useful because they standardize stimuli and increase replicability (Bendoly and Eckerd 2013). However, it can be argued that as these are hypothetical scenarios, some participants may have difficulty relating them to real world events and may respond in a socially desirable way as a due to this (Mlr et al. 2025). This can be especially problematic when attempting to review participants attitudes towards sensitive topics such as coercion and marital rape. Future research could control for this type of limitation with the use of virtual reality technology. This method has the potential to improve participant emotional engagement by allowing participants to engage with more realistic stimuli (Alok 2019; Green et al. 2025).

### 4.5 Further Research

Research reviewing the perceptions of marital rape could be expanded in the future by examining interactions among levels of force, perceptions of rape and perceptions of violence. Previous research suggests that individuals may not always view an incident as rape if the victim has been coerced or little to no violence has been used (Krulewitz and Payne 1978; Vandiver and Dupalo 2013; Hills et al. 2021). It is possible to argue that by gaining a better understanding of perceptions of marital rape, future researchers can create effective awareness programs for victims, courts, and the general public.

Future research could also investigate perceptions of marital rape further by using purposive sampling to ensure a proportion of the sample is made up of participants who identify as transgender or non‐binary. Recent research suggests that transgender and non‐binary individuals perceive gender roles as fluid and the traditional gender roles as a socially constructed concept (Nagoshi et al. 2012; Vijlbrief et al. 2020; J. Wilson et al. 2023). The inclusion of transgender and non‐binary participants could potentially assist with gaining further knowledge of how gender roles and gender identity may or may not have an impact on the perceptions of marital rape.

In addition to this, further research could examine how different levels of force used in marital rape scenarios can influence public perceptions of the severity of the situation and their decisions regarding the need for offender punishment. Additionally, the research could review how RMA and attitudes towards women mitigate these decisions. Previous research has indicated that individuals with high levels of sexism and RMA are more likely to assign offenders shorter sentences (Viki et al. 2004; C. J. Lilley 2021) and scenarios depicting violence received stricter punishments (Bergstrøm et al. 2017). This would help to gain a clearer idea of the public's perceptions of how marital rape cases should be sentenced during the trial process. In the U.K., Law Commission (2025) has reviewed the U.K. trial process and highlighted a greater need for improvements for judges and juries to understand rape myths, particularly those related to consent and sexual harm, to improve the treatment of complainants and fair trials within sexual offences cases. The recommendations have included the aim to protect defendants the right to a fair trial but emphasised the requirement for defence to present evidence without relying on myths. Law Commission (2025) has also recommended mandatory training for all legal practitioners on rape myths and for judges to educate juries on these myths.

## 5 Conclusion

Overall, the findings from the present study suggest that the level of force used does have an impact on the perception of rape in a marital rape scenario and these results add to the existing literature. However, further research is needed to gain a better understanding of factors which can influence public perceptions of marital rape and to increase our knowledge of why some myths are still accepted. These findings also support the Law Commission (2025) recommendation for all legal practitioners to receive mandatory training around the subject of rape myths and juries to be given education on these myths from judges. Based on these findings, it is possible to recommend that further awareness of legislation regarding rape, coercion, and marital rape is needed in the public domain.

## Author Contributions

**Leanne Hanney:** conceptualization, methodology, investigation, writing – original draft, writing – review and editing, project administration. **Amy Shelford:** methodology, data curation, writing – review and editing. **Andy Guppy:** conceptualization, writing – review and editing, formal analysis.

## Funding

The authors have nothing to report.

## Ethics Statement

Ethical approval was obtained from the Research Centre for Applied Psychology at the University of Bedfordshire. The procedures used in this study adhere to the tenets of the Declaration of Helsinki, and all participants gave their informed consent prior to data collection.

## Conflicts of Interest

The authors declare no conflicts of interest.

## Supporting information


Supporting Information S1

## Data Availability

The data that support the findings of this study will be available upon request from the lead author.

## References

[bsl70036-bib-0001] Agarwal, N. , S. M. Abdalla , and G. H. Cohen . 2022. “Marital Rape and Its Impact on the Mental Health of Women in India: A Systematic Review.” PLoS global public health 2, no. 6: e0000601. 10.1371/journal.pgph.0000601. 36962419 PMC10021972

[bsl70036-bib-0002] Agostini, E. , M. Schratz , and I. Eloff . 2024. Vignette Research: Research Methods. Bloomsbury Academic.

[bsl70036-bib-0003] Alok, S. 2019. “Vignette Methodology: An Illustration From Conflict Research.” In Qualitative Techniques for Workplace Data Analysis, edited by M. Gupta , M. Shaheen , and K. P. Reddy , 117–145. IGI Global.

[bsl70036-bib-0004] Angelone, D. J. , N. Cantor , T. Marcantonio , and M. Joppa . 2021. “Does Sexism Mediate the Gender and Rape Myth Acceptance Relationship?” Violence Against Women 27, no. 6–7: 748–765. 10.1177/1077801220913632. 32339090

[bsl70036-bib-0005] Angelone, D. J. , D. Mitchell , and L. Grossi . 2015. “Men’s Perceptions of an Acquaintance Rape: The Role of Relationship Length, Victim Resistance, and Gender Role Attitudes.” Journal of Interpersonal Violence 30, no. 13: 2278–2303. 10.1177/0886260514552448. 25287410

[bsl70036-bib-0006] Angelone, D. J. , D. Mitchell , and D. Smith . 2018. “The Influence of Gender Ideology, Victim Resistance, and Spiking a Drink on Acquaintance Rape Attributions.” Journal of Interpersonal Violence 33, no. 20: 3186–3210. 10.1177/0886260516635318. 26917569

[bsl70036-bib-0007] Barkan, S. , and G. Bryjak . 2011. Fundamentals of Criminal Justice: A Sociological View. Jones and Bartlett learning.

[bsl70036-bib-0008] Basile, K. C. 2002. “Prevalence of Wife Rape and Other Intimate Partner Sexual Coercion in a Nationally Representative Sample of Women.” Violence & Victims 17, no. 5: 511–524. 10.1891/vivi.17.5.511.33717. 12477095

[bsl70036-bib-0009] Bendoly, E. , and S. Eckerd . 2013. “Behavioural OM Experiments: Critical Inquiry Reawakening Practical Issues in Research.” In Behavioural Issues in Operations Management: New Trends in Design, Management, and Methodologies, edited by I. Giannoccaro , 1–22. Springer.

[bsl70036-bib-0010] Bergstrøm, H. , P. Evjetun , and M. Bendixen . 2017. “Punishment Justifications in Rape Cases: A Community Study.” Journal of Scandinavian Studies in Criminology and Crime Prevention 18, no. 2: 123–140. 10.1080/14043858.2017.1387451.

[bsl70036-bib-0011] Brown, S. , and E. Sleath . 2016. Research Methods for Forensic Psychologists: A Guide to Completing Your Research Project. Routledge.

[bsl70036-bib-0012] Burgin, R. 2019. “Persistent Narratives of Force and Resistance: Affirmative Consent as Law Reform.” British Journal of Criminology 59, no. 2: 296–314. 10.1093/bjc/azy043.

[bsl70036-bib-0013] Burt, M. R. 1980. “Cultural Myths and Supports for Rape.” Journal of Personality and Social Psychology 38, no. 2: 217–230. 10.1037/0022-3514.38.2.217. 7373511

[bsl70036-bib-0015] Cohen, J. 1988. Statistical Power Analysis for the Behavioural Sciences. Lawrence Erlbaum Associates, Publishers.

[bsl70036-bib-0016] DeGue, S. , D. DiLillo , and M. Scalora . 2010. “Are all Perpetrators Alike? Comparing Risk Factors for Sexual Coercion and Aggression.” Sexual Abuse 22, no. 4: 402–426. 10.1177/1079063210372140. 20693517

[bsl70036-bib-0017] Devine, F. , and S. Heath . 1999. Sociological Research Methods in Context. Bloomsbury Publishing.

[bsl70036-bib-0018] Eagly, A. H. , and A. Mladinic . 1989. “Gender Stereotypes and Attitudes Toward Women and Men.” Personality and Social Psychology Bulletin 15, no. 4: 543–558. 10.1177/0146167289154008.

[bsl70036-bib-0019] Edmond, W. A. , and T. D. Kennedy . 2016. An Applied Guide to Research Designs: Quantitative, Qualitative, and Mixed Methods. Sage publications.

[bsl70036-bib-0020] Edwards, K. M. , J. A. Turchik , C. M. Dardis , N. Reynolds , and C. A. Gidycz . 2011. “Rape Myths: History, Individual and Institutional‐Level Presence, and Implications for Change.” Sex Roles 65, no. 11–12: 761–773. 10.1007/s11199-011-9943-2.

[bsl70036-bib-0021] Evans, A. N. , and B. J. Rooney . 2008. Methods in Psychological Research. Sage Publications, Inc.

[bsl70036-bib-0022] Fakunmoju, S. B. , T. Abrefa‐Gyan , and N. Maphosa . 2019. “Confirmatory Factor Analysis and Gender Invariance of the Revised IRMA Scale in Nigeria.” Affilia 34, no. 1: 83–98. 10.1177/0886109918803645.

[bsl70036-bib-0023] Faul, F. , E. Erdfelder , A.‐G. Lang , and A. Buchner . 2007. “G*Power 3: A Flexible Statistical Power Analysis Program for the Social, Behavioral, and Biomedical Sciences.” Behavior Research Methods 39, no. 2: 175–191. 10.3758/bf03193146. 17695343

[bsl70036-bib-0024] Ferro, C. , J. Cermele , and A. Saltzman . 2008. “Current Perceptions of Marital Rape: Some Good and Not‐so‐Good News.” Journal of Interpersonal Violence 23, no. 6: 764–779. 10.1177/0886260507313947. 18272725

[bsl70036-bib-0025] Filip, O. L. , Popp, L. E. 2021. “Psychosocial Implications of Marital Rape.” In MATEC Web of Conferences, MATEC Web of Conferences Vol. 342, 10004. EDP Sciences. 10.1051/matecconf/202134210004.

[bsl70036-bib-0026] Frese, B. , M. Moya , and J. L. Megías . 2004. “Social Perception of Rape: How Rape Myth Acceptance Modulates the Influence of Situational Factors.” Journal of Interpersonal Violence 19, no. 2: 143–161. 10.1177/0886260503260245. 15005999

[bsl70036-bib-0027] Green, A. D. , A. Clark , J. Rechdan , and A. Guppy . 2025. “Virtual Reality Provides an Eyewitness Experience That is Similar to Real Life.” Applied Cognitive Psychology 39, no. 4: e70099. 10.1002/acp.70099.

[bsl70036-bib-0028] Hellier, E. J. 1998. “Within‐Subject Designs.” In Laboratory Psychology: A Beginner’s Guide, edited by J. Nunn , 39–57. Psychology Press Ltd.

[bsl70036-bib-0029] Hill, S. , and T. C. Marshall . 2018. “Beliefs About Sexual Assault in India and Britain Are Explained by Attitudes Toward Women and Hostile Sexism.” Sex Roles 79, no. 7–8: 421–430. 10.1007/s11199-017-0880-6. 30319168 PMC6156762

[bsl70036-bib-0030] Hills, P. J. , M. Pleva , E. Seib , and T. Cole . 2021. “Understanding How University Students Use Perceptions of Consent, Wantedness, and Pleasure in Labeling Rape.” Archives of Sexual Behavior 50, no. 1: 247–262. 10.1007/s10508-020-01772-1. 32642811 PMC7878243

[bsl70036-bib-0031] Home Office . 2022. Controlling or Coercive Behaviour: Statutory Guidance, What the Police and Organisations Should Do to Keep Victims Safe. Home Office. https://assets.publishing.service.gov.uk/media/6267c429e90e0716982a3250/ContCoerBehavStatGuid_V3‐_10‐04‐22_.pdf.

[bsl70036-bib-0032] Jaffe, A. E. , I. Cero , and D. DiLillo . 2021. “The# Metoo Movement and Perceptions of Sexual Assault: College Students’ Recognition of Sexual Assault Experiences Over Time.” Psychology of violence 11, no. 2: 209–218. 10.1037/vio0000363. 34970465 PMC8713172

[bsl70036-bib-0033] Jenkins, K. 2021. “Rape Myths: What Are They and What Can We Do About Them?” Royal Institute of Philosophy Supplement 89: 37–49. 10.1017/S1358246121000126.

[bsl70036-bib-0034] Krulewitz, J. E. , and E. J. Payne . 1978. “Attributions About Rape: Effects of Rapist Force, Observer Sex and Sex Role Attitudes.” Journal of Applied Social Psychology 8, no. 4: 291–305. 10.1111/j.1559-1816.1978.tb00784.x.

[bsl70036-bib-0035] Kuriakose, M. 2019. “Attitudes Towards Marital Rape: A Cross‐Cultural Study Between Young Adults in the United Kingdom and India.” International Journal of Indian Psychology 7: 308–321.

[bsl70036-bib-0036] Lagdon, S. , J. A. Jordan , P. Devine , M. A. Tully , C. Armour , and C. Shannon . 2023. “Public Understanding of Coercive Control in Northern Ireland.” Journal of Family Violence 38, no. 1: 39–50. 10.1007/s10896-021-00355-5. 35035065 PMC8744385

[bsl70036-bib-0037] Law Commission . 2025. Evidence in Sexual Offences Prosecutions: A Final Report. https://cloud‐platform‐e218f50a4812967ba1215eaecede923f.s3.amazonaws.com/uploads/sites/54/2025/07/ESOP‐Final‐Report‐July‐2025.pdf.

[bsl70036-bib-0038] Leeuw, F. L. , and H. Schmeets . 2016. Empirical Legal Research A Guidance Book for Lawyers, Legislators and Regulators. Edward Elgar Publishing Limited.

[bsl70036-bib-0039] Leon, C. M. , and E. Aizpurua . 2024. “Judging the Severity of Intimate Partner Violence Against Women: The Role of Situational Factors and Respondents’ Characteristics in Public Opinion.” Feminist Criminology 19, no. 2: 130–153. 10.1177/15570851231216920.

[bsl70036-bib-0040] Lilley, C. , D. Willmott , D. Mojtahedi , and D. Labhardt . 2023. “Intimate Partner Rape: A Review of Six Core Myths Surrounding Women’s Conduct and the Consequences of Intimate Partner Rape.” Social Sciences 12, no. 1: 34. 10.3390/socsci12010034.

[bsl70036-bib-0041] Lilley, C. J. 2021. Juror Decision‐Making Within Intimate Partner Rape: Examining the Relationship Between Modern Rape Myth Beliefs, Legal Attitudes and Personality Traits upon Verdict Decisions. Doctoral dissertation, University of Huddersfield.

[bsl70036-bib-0042] Mahoney, P. , and L. M. Williams . 1998. “Sexual Assault in Marriage: Prevalence, Consequences, and Treatment of Wife Rape.” In Partner Violence: A Comprehensive Review of 20 Years of Research, edited by J. L. Jasinski and L. M. Williams , 113–162. Sage.

[bsl70036-bib-0043] Martin, E. K. , C. T. Taft , and P. A. Resick . 2007. “A Review of Marital Rape.” Aggression and Violent Behavior 12, no. 3: 329–347. 10.1016/j.avb.2006.10.003.

[bsl70036-bib-0044] McMahon, S. , and G. L. Farmer . 2011. “An Updated Measure for Assessing Subtle Rape Myths.” Social Work Research 35 (November): 71–81: History & inception. 10.1093/swr/35.2.71metoo.Movement.

[bsl70036-bib-0075] me too. Movement . 2025. “History & Inception.” https://metoomvmt.org/get‐to‐know‐us/history‐inception/.

[bsl70036-bib-0045] Mlr, U. A. , S. Joldan , V. Kumar , and S. Saini . 2025. International Handbook of Research Methods and Statistics. Readers Paradise.

[bsl70036-bib-0046] Nagoshi, J. L. , S. I. Brzuzy , and H. K. Terrell . 2012. “Deconstructing the Complex Perceptions of Gender Roles, Gender Identity, and Sexual Orientation Among Transgender Individuals.” Feminism & Psychology 22, no. 4: 405–422. 10.1177/0959353512461929.

[bsl70036-bib-0047] Oates, J. , D. Carpenter , M. Fisher , et al. 2021. BPS Code of Human Research Ethics. British Psychological Society.

[bsl70036-bib-0048] Office for National Statistics . 2023. Sexual Offences Prevalence and Trends, England and Wales: Year Ending March 2022. Office for National Statistics website. https://www.ons.gov.uk/peoplepopulationandcommunity/crimeandjustice/articles/sexualoffencesprevalenceandtrendsenglandandwales/yearendingmarch2022.

[bsl70036-bib-0049] Oswald, D. L. , and B. L. Russell . 2006. “Perceptions of Sexual Coercion in Heterosexual Dating Relationships: The Role of Aggressor Gender and Tactics.” Journal of Sex Research 43, no. 1: 87–95. 10.1080/00224490609552302. 16817071

[bsl70036-bib-0050] Robinson, J. N. 2017. Marital Rape Perception and Impact of Force. CUNY Academic Works. https://academicworks.cuny.edu/jj_etds/10.

[bsl70036-bib-0051] Rollero, C. , and S. Tartaglia . 2019. “The Effect of Sexism and Rape Myths on Victim Blame.” Sexuality & Culture 23, no. 1: 209–219. 10.1007/s12119-018-9549-8.

[bsl70036-bib-0052] Russell, K. J. , and C. J. Hand . 2017. “Rape Myth Acceptance, Victim Blame Attribution and Just World Beliefs: A Rapid Evidence Assessment.” Aggression and Violent Behavior 37: 153–160. 10.1016/j.avb.2017.10.008.

[bsl70036-bib-0053] Serrano‐Montilla, C. , M. Garrido‐Macías , J. Sáez‐Díaz , and G. Sáez . 2025. “Assessing Police Attitudes Toward Intervention in Gender Violence: The Role of Training, Perceived Severity, and Myths About Intimate Partner Violence Against Women.” Journal of Family Violence 40, no. 2: 299–311. 10.1007/s10896-023-00605-8.

[bsl70036-bib-0054] Sharma, S. , and D. C. Mishra . 2021. “Marital Rape Criminalization: A Critical Study.” Ilköğretim Online 20: 2338–2347.

[bsl70036-bib-0055] Shoda, Y. 2004. “Individual Differences in Social Psychology: Understanding Situations to Understand People, Understanding People to Understand Situations.” In The Sage Handbook of Methods in Social Psychology, edited by A. T. Panter , C. Sansone , and C. C. Morfn , pp117–pp128. Sage Publications.

[bsl70036-bib-0056] Simonson, K. , and L. M. Subich . 1999. “Rape Perceptions as a Function of Gender‐Role Traditionality and Victim‐Perpetrator Association.” Sex Roles 40, no. 7–8: 617–634. 10.1023/a:1018844231555.

[bsl70036-bib-0057] Spence, J. T. , and E. D. Hahn . 1997. “The Attitudes Toward Women Scale and Attitude Change in College Students.” Psychology of Women Quarterly 21, no. 1: 17–34. 10.1111/j.1471-6402.1997.tb00098.x.

[bsl70036-bib-0058] Spence, J. T. , R. Helmreich , and J. Stapp . 1973. “A Short Version of the Attitudes Toward Women Scale (AWS).” Bulletin of the Psychonomic Society 2, no. 4: 219–220. 10.3758/bf03329252.

[bsl70036-bib-0059] Stommel, M. , and C. E. Wills . 2004. Clinical Research: Concepts and Principles for Advanced Practice Nurses. Lippincott Williams & Wilkins.

[bsl70036-bib-0060] Suarez, E. , and T. M. Gadalla . 2010. “Stop Blaming the Victim: A Meta‐Analysis on Rape Myths.” Journal of Interpersonal Violence 25, no. 11: 2010–2035. 10.1177/0886260509354503. 20065313

[bsl70036-bib-0061] Szekeres, H. , E. Shuman , and T. Saguy . 2020. “Views of Sexual Assault Following# Metoo: The Role of Gender and Individual Differences.” Personality and Individual Differences 166: 110203. 10.1016/j.paid.2020.110203.

[bsl70036-bib-0062] Tarzia, L. 2021. “‘It Went to the Very Heart of Who I Was as a Woman’: The Invisible Impacts of Intimate Partner Sexual Violence.” Qualitative Health Research 31, no. 2: 287–297. 10.1177/1049732320967659. 33118450

[bsl70036-bib-0063] The Law Commision . 1992. “Criminal Law: Rape Within a Marriage.” Retrieved November 15, 2022 from Gov.uk. https://assets.publishing.service.gov.uk/government/uploads/system/uploads/attachment_data/file/228746/0167.pdf.

[bsl70036-bib-0064] Utami, F. R. , and H. A. M. Haeranah . 2022. “Visum Et Repertum as Evidence in the Crime of Marital Rape.” Journal of Positive School Psychology 6: 1140–1149.

[bsl70036-bib-0065] Vandiver, D. M. , and J. R. Dupalo . 2013. “Factors That Affect College Students’ Perceptions of Rape: What Is the Role of Gender and Other Situational Factors?” International Journal of Offender Therapy and Comparative Criminology 57, no. 5: 592–612. 10.1177/0306624x12436797. 22436736

[bsl70036-bib-0066] Viejo, C. M. , and A. M. Sanz . 2020. “Between Coercion and Consent: A Study on Male Sexual Violence in Heterosexual Partner Relationship.” Masculinidades y cambio social 9: 235–260.

[bsl70036-bib-0067] Vijlbrief, A. , S. Saharso , and H. Ghorashi . 2020. “Transcending the Gender Binary: Gender Non‐Binary Young Adults in Amsterdam.” Journal of LGBT Youth 17, no. 1: 89–106. 10.1080/19361653.2019.1660295.

[bsl70036-bib-0068] Viki, G. T. , D. Abrams , and B. Masser . 2004. “Evaluating Stranger and Acquaintance Rape: The Role of Benevolent Sexism in Perpetrator Blame and Recommended Sentence Length.” Law and Human Behavior 28, no. 3: 295–303. 10.1023/b:lahu.0000029140.72880.69. 15264448

[bsl70036-bib-0069] Walker, D. G. , Y. Teerawattananon , R. Anderson , and G. Richardson . 2010. “Generalisability, Transferability, Complexity and Relevance.” In Evidence‐Based Decisions and Economics: Health Care, Social Welfare, Education and Criminal Justice, edited by I. Shemilt , M. Mugford , L. Vale , K. Marsh , and C. Donaldson , 56–66. Wiley‐Blackwell.

[bsl70036-bib-0070] West, C. M. 2002. “Black Battered Women: New Directions for Research and Black Feminist Theory.” In Charting a New Course for Feminist Psychology, edited by J. C. Chrisler , L. H. Collins , and M. R. Dunlap , 216–237. Praeger.

[bsl70036-bib-0071] Wilson, C. 2024. Conducting Applied Psychological Research: A Practical Guide. Open University Press.

[bsl70036-bib-0072] Wilson, J. , C. Sargeant , N. Jago , and S. Wright . 2023. “Co‐creating a New Theory of Gender Beyond the Binary: A Delphi Study in the United Kingdom.” Journal of LGBT Youth 21, no. 3: 1–25. 10.1080/19361653.2023.2202664.

[bsl70036-bib-0073] Woolley, M. L. 2007. “Marital Rape: A Unique Blend of Domestic Violence and Non‐Marital Rape Issues.” Hastings Women's LJ 18: 269.

[bsl70036-bib-0074] World Health Organization . 2021. Violence Against Women. World Health Organization. https://www.who.int/news‐room/fact‐sheets/detail/violence‐against‐women.

