# Supplementary material for: Public Perceptions of Marital Rape: Does Level of Force Used Have an Impact?
Source: Behav Sci Law. 2026 Jan 10;44(2):224–34. doi: 10.1002/bsl.70036 (PMC13053914; doi:10.1002/bsl.70036)
Supplement: Supplementary file 1 — Supporting Information S1 [file BSL-44-224-s001.docx]

Appendix 1: Robinson (2017) Marital rape post vignette survey

1. To what extent was Barbara in control of the situation?

0 – Not at all 1- Very little 2- Somewhat 3-To a great extent 4- Completely

2. To what extent was the incident influenced by Barbara’s lack of responsibility for the situation?

0 – Not at all 1- Very little 2- Somewhat 3-To a great extent 4- Completely

3. To what extent was the incident influenced by Barbara’s desire for intercourse?

0 – Not at all 1- Very little 2- Somewhat 3-To a great extent 4- Completely

4. To what extent did Barbara lead John on?

0 – Not at all 1- Very little 2- Somewhat 3-To a great extent 4- Completely

5. To what extent was the situation influenced by John’s misunderstanding of Barbara’s behavior or desires?

0 – Not at all 1- Very little 2- Somewhat 3-To a great extent 4- Completely

6. To what extent was the situation influenced by John’s inability to stop the incident?

0 – Not at all 1- Very little 2- Somewhat 3-To a great extent 4- Completely

7. To what extent was the situation influenced by John’s excessive sex drive?

0 – Not at all 1- Very little 2- Somewhat 3-To a great extent 4- Completely

8. To what extent was the situation influenced by John’s responsibility?

0 – Not at all 1- Very little 2- Somewhat 3-To a great extent 4- Completely

9. To what extent would Barbara be psychologically damaged?

0 – Not at all 1- Very little 2- Somewhat 3-To a great extent 4- Completely

10. To what extent was John violating Barbara’s rights?

0 – Not at all 1- Very little 2- Somewhat 3-To a great extent 4- Completely

11. Would you characterize the intercourse as rape?

0 – Definitely not rape 1- Probably not rape 2- Possibly rape 3- Probably rape 4- Very probably rape 5. Definitely rape

12. To what extent was the incident violent?

0 – Not violent at all 1- Slightly violent 2- Moderately violent 3-Very Violent 4- Extremely violent

13. In your opinion, does John deserve punishment?

0 – Not at all 1- Possibly 2- Probably 3-Definitely

14. If you think that John deserves punishment, how severe should it be?

0 – Very light Punishment 1- Mild punishment 2- Moderate punishment 3- Harsh punishment 4- Very severe punishment

Appendix 2: Vignettes with British Residents – adapted from Robinson (2017) and Simonson and Subich (1999).

Marital rape Subtle Force:

One night Mary and Alan; both Leeds residents attending university, went on a dinner date. Mary and Alan have been married for 3 years and both grew up in Leeds. Following the dinner, Mary and Alan went back to their apartment and watched a late night television series. While watching the series, Alan put his arm around Mary and asked her if she wanted to have sex. Mary told him “no” but Alan ignored her response. Alan embraced her, lifted her skirt and completed the act of intercourse.

Marital rape Moderate Force:

One night Linda and Mario; both Brighton residents attending university, went on a dinner date. Linda and Mario have been married for 3 years and both grew up in Brighton. Following the dinner, Linda and Mario went back to their apartment and watched a late night television series. While watching the series, Mario put his arm around Linda and asked her if she wanted to have sex. Linda told him “no” but Mario ignored her response. Mario grabbed her shoulders, pinned her down and completed the act of intercourse.

Marital rape Extreme Force:

One night Leticia and John; both Leicester residents attending university, went on a dinner date. Leticia and John have been married for 3 years and both grew up in Leicester. Following the dinner, Leticia and John went back to their apartment to catch a late night television series. While watching the series, John put his arm around Leticia and asked her if she wanted to have sex. Leticia told him “no” but John ignored her response. John grabbed her by the hair, threw her to the couch, forced himself on top of her and completed the act of intercourse.
